# Reducing length of stay for acute diabetic foot episodes: employing an extended scope of practice podiatric high-risk foot coordinator in an acute foundation trust hospital

**DOI:** 10.1186/1757-1146-6-47

**Published:** 2013-12-11

**Authors:** Matthew J Cichero, Virginia M Bower, Tom P Walsh, Ben J Yates

**Affiliations:** 1 Great Western Hospitals NHS Foundation Trust, Great Western Hospital, Marlborough Road, Swindon, UK; 2 School of Surgery, Faculty of Medicine, Dentistry and Health Sciences, The University of Western Australia, Crawley, Australia; 3 Department of Podiatry, Repatriation General Hospital, Mitcham, South Australia, Australia

**Keywords:** Diabetic foot, Podiatry, Inpatients, Multidisciplinary communication

## Abstract

**Background:**

To enhance the acute management of people with diabetic foot disease requiring admission, an extended scope of practice, podiatric high-risk foot coordinator position, was established at the Great Western Hospital, Swindon in 2010. The focus of this new role was to facilitate more efficient and timely management of people with complex diabetic foot disease. The aim of this project was to investigate the impact of the podiatric high-risk foot coordinator role on length of stay, rate of re-admission and bed cost.

**Method:**

This study evaluated the difference in length of stay and rate of re-admission between an 11- month pre-pilot period (November 2008 to October 2009) and a 10-month pilot period (August 2010 to June 2011). The estimated difference in bed cost between the pre-pilot and pilot audits was also calculated. Inclusion criteria were restricted to inpatients admitted with a diabetic foot ulcer, gangrene, cellulitis or infection as the primary cause for admission. Eligible records were retrieved using ICD-10 (V9) coding via the hospital clinical audit department for the pre-pilot period and a unique database was used to source records for the pilot phase.

**Results:**

Following the introduction of the podiatric high-risk foot coordinator, the average length of stay reduced from 33.7 days to 23.3 days (mean difference 10.4 days, 95% CI 0.0 to 20.8, *p* = 0.050). There was no statistically significant difference in re-admission rate between the two study periods, 17.2% (95% CI 12.2% to 23.9%) in the pre-pilot phase and 15.4% (95% CI 12.0% to 19.5%) in the pilot phase (*p* = 0.820). The extrapolated annual cost saving following the implementation of the new coordinator role was calculated to be £234,000 for the 2010/2011 year.

**Conclusions:**

This audit found that the extended scope of practice coordinator role may have a positive impact on reducing length of stay for diabetic foot admissions. This paper advocates the role of a podiatric high-risk foot coordinator utilising an extended scope of practice model, although further research is needed.

## Background

Diabetic foot disease is characterised by peripheral arterial disease, peripheral neuropathy, ulceration, infection, joint deformity, joint destruction and amputation [1,2]. Management of the diabetic foot may include regular outpatient consultation; frequent presentation to accident and emergency; extended antimicrobial therapy; prolonged hospitalisation and emergency amputation — all of which have a significant personal and financial impact on the individual and society [3].

The inpatient management of the diabetic foot is of equal importance to outpatient management as patients admitted for an acute diabetic foot condition are particularly vulnerable to poor outcomes, with emergency management often necessary. The length of stay for patients with diabetes can be prolonged [4-6], with various factors compounding the difficulty in resolving foot complaints. If investigations, interventions, consultations and care planning are not coordinated during an inpatient stay by appropriately skilled and experienced health professionals, it is our belief that length of stay is extended, re-admission is more likely, and poorer clinical outcomes expected.

Over the past 20 years, evidence has accumulated in support of the multidisciplinary team model for the prevention and management of diabetic foot complications in the outpatient setting, although little focus has been given to the inpatient setting [7-11]. Within the clinical management of diabetes, there is recognition that coordination of care for inpatients is fundamental, however this is yet to be formally recognised within clinical guidelines. The National Health and Medical Research Council in Australia has produced guidelines which identify that there is a general need for improved co-ordination and multi-disciplinary care planning, however these documents fail to provide any specific detail on inpatient management [12,13]. Similar recognition has occurred in the United Kingdom (UK). The National Institute of Clinical Excellence (NICE) has sought to address this gap in diabetic foot management with the publication of their 2011 diabetic foot management guidelines [14]. The NICE guidelines recommend that inpatient management of the diabetic foot requires particular attention. Specifically, the NICE guidelines strongly recommend that one health professional should be responsible for coordinating inpatient care between specialists, the patient and other health professionals. The guideline advocates that this pivotal professional oversee the multidisciplinary coordination of care, schedule relevant interventions and investigations, and ensure appropriate and timely discharge planning.

In the UK and Australia, a medical practitioner would commonly fill the role described by the NICE guidelines, as many of the responsibilities would be outside the scope of practice for a registered podiatrist working in the public health sector. Functions and procedures such as admission of patients requiring inpatient care; requesting and interpreting complex radiological imaging; and surgical debridement are currently beyond the scope of practice for podiatrists employed in the public health system in Australia. Internationally, there are many examples of professions seeking and acquiring pathways to extended scope of practice [15-18]. The adoption of the nurse practitioner model is one such example where nurses have successfully acquired an extension to their scope of practice through the acquisition of higher academic qualifications and vocational training [19]. The example of podiatric surgery in Australia and the UK is another case where advanced degree qualification and vocational training advanced the scope of practice for podiatrists, further integrating podiatry into the existing medical model [20]. Within the international podiatry community, there is a long held acknowledgement that high-risk foot care is a specialist branch of the profession. In the absence of a regulated post-registration career path option for this discipline, there remains no official recognition of this role via specialist registration in Australia or the UK [21].

With this background in mind, the development of the diabetic foot team at Great Western Hospital, Swindon, UK has been evolving since 2004. Initially the team consisted of a vascular surgeon, endocrinologist, a podiatric surgeon, and part-time diabetes podiatrist. Patients were managed by the individual intra-disciplinary teams with ad-hoc cross referrals made to other specialists as deemed necessary. Over time the team recognised that there were many inadequacies with this clinical structure and sought ways for improvement. A weekly multidisciplinary ward round was commenced in 2009. The team also identified that in order to facilitate improved coordination and efficiency of inpatient management, a diabetic foot coordinator was required full time, five days per week. It was envisaged that this role would facilitate appropriate and timely investigations and management in an attempt to enhance patient care.

The Great Western Hospital was keen to recruit into the coordinator role a podiatrist with formalised post-graduate training. A Masters level postgraduate qualification (i.e. a specialist podiatry-related Masters degree); training and experience in advanced imaging, haematological assessment and interpretation; and experience in managing the diabetic foot were identified as desirable selection criteria. The job description was essentially a hybrid of the UK National Health Service job description for a podiatric surgeon, although surgical training was not an essential criterion for the new position.

In August 2010, The Great Western Hospital initiated the new position, which was held by a podiatric surgical registrar who had several years of hospital high-risk foot experience. This new position was on-call during office hours five days a week. The aim of this pilot position was to evaluate the benefits of having a podiatric coordinator to oversee the management of patients admitted with acute diabetic foot complications. The extended scope of practice role description of the podiatric high-risk foot coordinator can be found in Table 1. The Great Western Hospital aimed to create a position that would bring together and enhance the performance of the existing specialist team. It was intended that the role would provide the best possible coordinated care to the patient, free of professional scope of practice barriers, which render many multidisciplinary teams less effective.

**Table 1:** Role description of the extended scope of practice podiatric high-risk foot coordinator

**Primary role of the podiatric high risk foot coordinator**	**Specific roles of the podiatric high risk foot coordinator**
Patient admission.	• Selection of admitting discipline and processing.
Inpatient management.	• Requesting and interpreting haematological analyses.
	• Requesting and performing deep tissue samples for microbiology and histopathology.
	• Requesting and interpreting radiological imaging including plain x-ray, ultrasonography, CT, CTPet and MRI.
	• Requesting and interpreting specialised vascular imaging including MRI angiography and duplex ultrasonography.
	• Coordinating inter-specialist, nursing and allied health referral.
	• Liaison with microbiology for antibiotic management.
	• Bedside wound debridement.
	• Participating in multidisciplinary vascular and endocrinology team meetings.
Emergency and prophylactic surgery. (Not essential for the role, but advantageous)	• Coordinating and performing surgical procedures.
	• Requesting and arranging peri-operative care in conjunction with junior medical staff; including sliding scales, blood and platelet transfusions etc.
Discharge planning and outpatient management.	• Outpatient podiatric surgery clinics for assessment and planning of elective prophylactic diabetic foot surgery and ongoing management of acute and chronic Charcot foot complications.

The aim of this project was to evaluate a new model of care involving an extended scope of practice podiatric high-risk foot coordinator.

## Methods

The study was an audit designed to evaluate the effectiveness of the podiatric high-risk foot coordinator position. Effectiveness was measured by a change in length of stay for patients admitted with acute diabetic foot complications before and after the implementation of the podiatric high-risk foot coordinator position. The authors acknowledge that length of stay is only one variable that may be associated with a modified model of care, so to complement the length of stay data, re-admission and cost data were also collected and analysed for comparison between the two data collection periods. Ethics approval was not required to undertake this study as it was classified as a clinical audit.

A retrospective medical record audit was performed at Great Western Hospital Primary Care Trust between 1st November 2008 and the 1st of October 2009 to determine the average length of stay for eligible diabetic foot admissions for the pre-pilot period. Inclusion criteria for the audit were restricted to inpatients admitted with a diabetic foot ulcer, gangrene, cellulitis or infection as the primary cause for admission. Eligible records were retrieved using ICD-10 coding via the hospital’s clinical audit department. The ICD-10 codes used to identify eligible records are displayed in Table 2.

**Table 2:** ICD-10 (V9) coding used to identify eligible records

**Diagnosis**	**ICD-10 Coding**
Diabetes	E10. – E14.
Cellulitis	L03.0 – L03.1
Gangrene	E10.5 – E11.5
Subsidiary for gangrene	R02X

Nine months after the commencement of the podiatric high-risk foot coordinator position (2nd August 2010 to 30th June 2011), the pilot audit cycle was completed. Pilot period data was identified using the same inclusion and exclusion criteria as the pre-pilot period data. Pilot period data was retrieved from a unique database established by the podiatric coordinator for the purpose of this audit.

Measuring the quality of inpatient diabetes care is difficult and a variety of outcome measures have been previously used including quantitative and qualitative measures. Length of stay is a quantitative outcome measure that is well established, routinely measured and considered to be of economic importance. Length of stay has been previously utilised to measure the impact of new service delivery models in diabetes care [4-6] and has achieved acceptance as a reasonable surrogate to assess quality of care. For this reason we chose to use length of stay in this study as our primary outcome measure of success. Length of stay was defined as the duration of a single episode of hospitalisation, and no re-admission within 48 hours of discharge. Length of stay was calculated by subtracting the day of admission from day of discharge. Patients entering and leaving hospital on the same day were allocated a length of stay of one. Re-admission was defined as patients admitted for another episode of diabetic foot infection after 48 hours but no more than six months post initial discharge. The re-admission needed to be directly associated with the location of the preceding foot infection.

Statistical analyses must account for the grouping of the data due to multiple responses from patients both within and between sampling periods. Such grouping may violate the assumptions of classical statistical inference that underlie, for example, t-tests for continuous data and chi-square for dichotomous data. Consequently, we have applied the extensions of generalised linear model theory [22], modelling that permits the comprehensive incorporation of both continuous and dichotomous data to mixed-effects models [23], which permit grouping of the data. Statistical testing in mixed-effects models is carried out by likelihood-ratio tests with a chi-square distribution, where *p*-values are quoted throughout; *p*-values of ≤0.05 were considered statistically significant.

The estimated difference in cost between the pre-pilot audit and the pilot audit were calculated using the average bed cost of UK£250.00 per bed day provided by the Great Western Hospital Finance Department.

## Results

There were 34 episodes identified in the initial audit that met the inclusion criteria. Seventy-five episodes met the inclusion criteria for the pilot period. The average length of stay for the pre-pilot phase was 33.7 days compared with 23.3 days in the pilot phase (mean difference 10.4 days, 95% CI 0.0 to 20.8, *p* = 0.050).

In the pre-pilot phase, there were 5 re-admissions among 29 patients, giving a 17.2% re-admission rate (95% CI 12.2% to 23.9%). In the pilot period, there were 10 re-admissions among 65 patients, giving a 15.4% re-admission rate with a 95% confidence interval of (12.0% to 19.5%). The difference between rates was not statistically significant (*chi-square* = 0.05, *d.f.* = 1, *p* = 0.820).

The extrapolated annual cost saving following the implementation of the podiatric high-risk foot coordinator and the resulting reduction in length of stay was calculated to be UK£234,000 for the 2010/2011 year. Table 3 provides a breakdown of the figures used to calculate this cost saving.

**Table 3:** Extrapolated annual cost saving in UK pounds between pre-pilot audit and pilot audit

	**Pre-pilot audit 2008/2009**	**Pilot audit 2010/2011**	**Difference/saving**
No. of episodes	34	75	
Data collection period in months	11	10	
Extrapolated episodes full year equivalent	37	90	
Average LOS	33.7	23.3	10.4
Average LOS bed cost [LOS x cost per bed day (£250.00)]	£8,425.00	£5,825.00	£2,600.00
Extrapolated annual saving (LOS bed cost difference pre-pilot/post pilot) x (extrapolated episodes pilot)	(£2,600.00 x 90) = £234,000.00		

## Discussion

Length of stay is reported to be a reasonable surrogate to measure quality of hospital diabetes care [4-6] and was used in the current study to assess the impact of the new podiatric high-risk foot coordinator role. Our audit identified a significant reduction in length of stay for diabetic foot admissions following the introduction of our new model of care. Our study findings are consistent with previous studies, which have used length of stay to measure the impact of new models of care for inpatient delivery of diabetes care. Flanagan et al. reported a significant reduction in average length of stay of inpatients with diabetes over a five-year period following the introduction of a specialised inpatient diabetes team at their hospital [4]. In the Flanagan et al. study length of stay was reported to have reduced most significantly for emergency admissions. This is a useful comparison with the results of the current study, which also involved acute diabetic foot admissions.

Re-admission of patients with diabetic foot complications is a well-recognised phenomenon. While critics may suggest that reducing length of stay will increase the rate of re-admission, there is little evidence in the literature to support this claim [24]. Our study found that there was no increase in re-admission rate among cases included in this study. The authors acknowledge that re-admission is a complex issue with many potential confounding variables and that there is a need for more controlled studies before an association can be confidently made between length of stay and re-admission.

Both the outpatient and inpatient costs attributed to diabetic foot complications are high, however it is well acknowledged that inpatient care contributes the greatest cost due to the intensity and complexity of care at this level [25]. Stockl and colleagues estimated that 77% of the overall cost associated with the treatment of diabetic foot ulcers included in their study was attributed to hospitalisation [26]. Daultrey and colleagues report that length of stay in the National Health Service is increased for patients with diabetes due to the complexity of the disease and the lack of specialised inpatient care [27]. The same study reports that increased length of stay contributes to 80,000 excess bed days per year across England.

Consistent with previous findings [26,27], our study found that a cost saving of £234,000.00 per annum was associated with a reduction in the length of stay. We acknowledge that our results cannot be directly compared with previous studies, as we were unable to identify previous published research that measured an association between bed cost and new models of care such as the podiatric high-risk foot coordinator. We can, however, suggest that our new model of care has had an indirect impact on bed cost by achieving a reduction in length of stay.

We appreciate the importance of appropriate management in the outpatient setting, however we wish to emphasise the crucial and frequently neglected role of coordinated care at the inpatient level. Diabetic foot infection requiring admission is considered a medical emergency and may be accompanied by a threat to a limb or life [28-31]. An acute diabetic foot admission requires rapid and well-coordinated care to ensure a successful clinical outcome for the patient [32]. Previous authors have identified the variability in the assessment and management of patients admitted for acute diabetic foot complications [33]. Lawrence et al. [34] found that even simple recognition of previous amputation and inspection of pulses are often not recorded in medical records. The need for expeditious and high-level clinical care for inpatients was one of the primary motivations for the Great Western Hospital to initiate the new role described in this article. The hospital recognised that it was necessary for the new role to have a coordinating responsibility and be equipped with a broad scope of practice to facilitate the most timely and effective care of inpatients.

The literature supports the role of a single professional to coordinate inpatient care. There is evidence in the literature that a podiatrist led team is successful in reducing amputation rates [35,36]. The high-risk foot coordinator is, however, only as effective as the members of the group. All group members must be allowed to make contributions and perform the needed functions of the group free of inhibitions and professional boundaries. An effective coordinator must be a good leader with the skills to collate opinions and prepare a mutually agreeable care plan without any one discipline attempting to dominate the team.

We found that the success of the inpatient team relied on having the podiatric high-risk foot coordinator present at the multidisciplinary team meetings as well as reviewing patients at the time of admission and coordinating the ward rounds. With this level of involvement the coordinator was able to facilitate key investigations and interventions promptly without delay. Opinions from the various non-team specialists were also more easily obtained due to a change from traditional methods of communication which relied on referral forms that could take days to be answered.

We found that the surgical skills the incumbent podiatrist brought to this new role were beneficial but not essential to the success of the position. We would like to emphasise that the results achieved in this audit were not dependent on the surgical skill set of the podiatrist who filled the role, as the number of procedures performed by high-risk foot coordinator was small. A consultant podiatric surgeon performed the majority of the surgical work during the audit period.

We believe that any future development of the new role described in this paper, needs to be underpinned by structured post-graduate training. In the past, innovative specialist areas in podiatry relied on charismatic individuals to form relationships and ‘gentlemen’s agreements’ [21]. However, the authors of this paper and other prominent commentators on this topic advocate that the acute diabetic foot demands an appropriately trained and recognised, expert care provider. A Masters level qualification supported by internships in medicine, pharmacology, microbiology, surgery and radiology combined with independent prescribing qualifications would be a suitable benchmark to establish.

The authors acknowledge that there are a number of significant limitations of this study. The pre-pilot period data was collected retrospectively while the pilot data was collected prospectively. The pre-pilot data is not as robust as the pilot data due to the errors associated with retrospective data analysis. The authors acknowledge this and every effort was made to match the data between the two cohorts to ensure the most reasonable comparison could be made. Cross-referencing of patients identified in the retrospective medical record audit was performed by manually searching the patient record to confirm that the eligible ICD-10 code was recorded correctly. In addition, a mathematical assumption for the distribution of both samples was made regarding the independence and identical nature of the two sets of data when calculating the average length of stay.

The authors also acknowledge that there are seasonal variations in acute diabetic foot presentations to hospitals. The seasons varied between the pre-pilot and pilot data collection periods in this study, although there were ten months in common and therefore, it is unlikely that this issue had a significant impact on the results. The period of data collection varies between the pre-pilot and pilot periods by one month. While every effort was made by the authors to collect one calendar year of data for each group this was not possible due to administrative requirements of the hospital. The data collection period for the pilot period was influenced by funding and reporting requirements of the hospital.

The difference in sample size between the pre-pilot and the pilot periods is noted by the authors and two explanations are made for this variation. The unreliable nature of retrospective data audit is a likely reason for lower numbers in the pre-pilot group despite the best efforts of the authors to identify as many eligible records as possible. An additional explanation is the occurrence of the ‘honeymoon phenomenon’ that often occurs with the establishment of new services whereby referrals initially increase with increased awareness among hospital staff of the benefit the service can bring. It is also reasonable to assume that patients were identified and coded more accurately on commencement of the podiatric high-risk foot coordinator in the emergency department of the hospital.

This study has revealed some interesting findings, which we believe would benefit from further investigation. Future parallel-group randomised trials would be of value to evaluate the true impact of high risk foot coordinators on the outcomes we have measured in our study. Any future prospective research on this topic would benefit from an expansion of the outcome measures being evaluated. In addition, we believe that the complex nature of the diabetic foot condition lends itself to further exploration using mixed method research. Qualitative data examining the patient perspective of different models of diabetic foot care would be an invaluable contribution to further research on this topic. Qualitative enquiry would compliment and expand on quantitative methods, which are well acknowledged to be limited in the depth and breadth of knowledge they deliver to the researcher.

## Conclusion

This study supports the recommendations made by NICE that a coordinator for acute diabetic foot admissions is a valuable asset to the hospital diabetes team. The study found that the implementation of the new coordinator position at the Great Western Hospital was associated with a reduced length of stay of diabetic foot admissions during the study period. The coordinator role also ensured timely and appropriate discharge planning, which should in turn, prevent future admissions and unnecessary major amputations. We believe that there is a demand for specialisation in the high-risk foot, which can be achieved with the establishment of a podiatric high-risk foot coordinator role. Although further research is needed, the authors anticipate that the results of this audit and the ideas discussed in this article may generate debate on this topic.

## Competing interests

The authors declare that they have no competing interests.
